# NMR assignment of the conserved bacterial DNA replication protein DnaA domain IV

**DOI:** 10.1007/s12104-024-10206-1

**Published:** 2024-10-04

**Authors:** Alexander Nguyen Abrams, Geoff Kelly, Julia Hubbard

**Affiliations:** 1 Newcastle University Biosciences Institute, Newcastle University, Newcastle Upon Tyne, NE2 4AH UK; 2 The Medical Research Council Biomedical NMR Centre, The Francis Crick Institute, 1 Midland Road, London, NW1 1AT UK

**Keywords:** DnaA, DNA replication, Initiation, DNA-binding

## Abstract

Chromosomal replication is a ubiquitous and essential cellular process. In bacteria, the master replication initiator DnaA plays a key role in promoting an open complex at the origin (*oriC*) and recruiting helicase in a tightly regulated process. The C-terminal domain IV specifically recognises consensus sequences of double-stranded DNA in *oriC*, termed DnaA-boxes, thereby facilitating the initial engagement of DnaA to *oriC*. Here, we report the ^13^Cβ and backbone ^1^H, ^15^N, and ^13^C chemical shift assignments of soluble DnaA domain IV from *Bacillus subtilis* at pH 7.6 and 298 K.

**Supplementary Information:**

The online version contains supplementary material available at 10.1007/s12104-024-10206-1.

## Biological context

Genomic replication plays a universal and essential role in cell proliferation. In bacteria, genetic material is contained within circular chromosomes and replication proceeds from a single origin (*oriC*) bidirectionally (O’Donnell et al. 2013). Initiation involves the loading of helicase which unwinds DNA before duplication. Replication initiation at *oriC* is tightly coordinated to cellular growth rate to ensure a singular occurrence during the cell cycle (Skarstad and Katayama 2013; Wolanski et al. 2015; Riber et al. 2016). In *Bacillus subtilis*, five proteins are sequentially recruited to *oriC* to initiate replication: the master initiator DnaA; the helicase loaders DnaD, DnaB, and DnaI; and the replicative helicase DnaC (Smits et al. 2010). The ubiquitous DnaA initially binds to *oriC* to recruit the helicase loaders as well as promoting the formation of an open complex to facilitate helicase loading in a tightly regulated process (Hwang and Kornberg 1992; Katayama et al. 2017).

In *B.* subtilis, DnaA is a 50.9 kDa protein comprising four domains across 446 amino acids. DnaA recognises and binds to a 9-bp consensus sequence, 5’-TTATnCACA-3’, termed the DnaA-box (Fuller et al. 1984; Roth and Messer 1995; Obita et al. 2002; Fujikawa et al. 2003; Yoshida et al. 2003). Multiple DnaA-boxes exist in *oriC* whereby upon recruitment, ATP-bound DnaA readily forms a right-handed helical head-to-tail filament that promotes unwinding of the duplex unwinding element (DUE) (Schaper and Messer 1995; Felczak and Kaguni 2004; Erzberger et al. 2006; Pelliciari et al. 2023). Out of the four domains, it is the C-terminal domain IV that recognises and binds to the DnaA-box. DnaA domain IV, which is 94 residues in length, adopts a fold comprising six α-helices and four loops (Erzberger et al. 2002; Fujikawa et al. 2003; Tsodikov and Biswas 2011; Pelliciari et al. 2023). Domain IV comprises a helix-turn-helix (HTH) motif recognising both the backbone and bases of the DnaA-box and conferring specificity (Sutton and Kagnni 1997; Blaesing et al. 2000). As part of the HTH motif, the α-helix between D413 and D430 inserts into the major-groove forming specific contacts to several bases. At the same time, a basic loop region between K375 and K381 contacts the minor-groove (Fujikawa et al. 2003; Pelliciari et al. 2023). N-terminal residues of the terminal α-helix have also been evidenced to engage the minor-groove (Pelliciari et al. 2023). Last, domain IV is connected to domain III by a short linker region thought to play a key role in swiveling domain IV with respect to domain III during the assembly of DnaA oligomers (Erzberger et al. 2002; Shimizu et al. 2016).

**Fig. 1:**
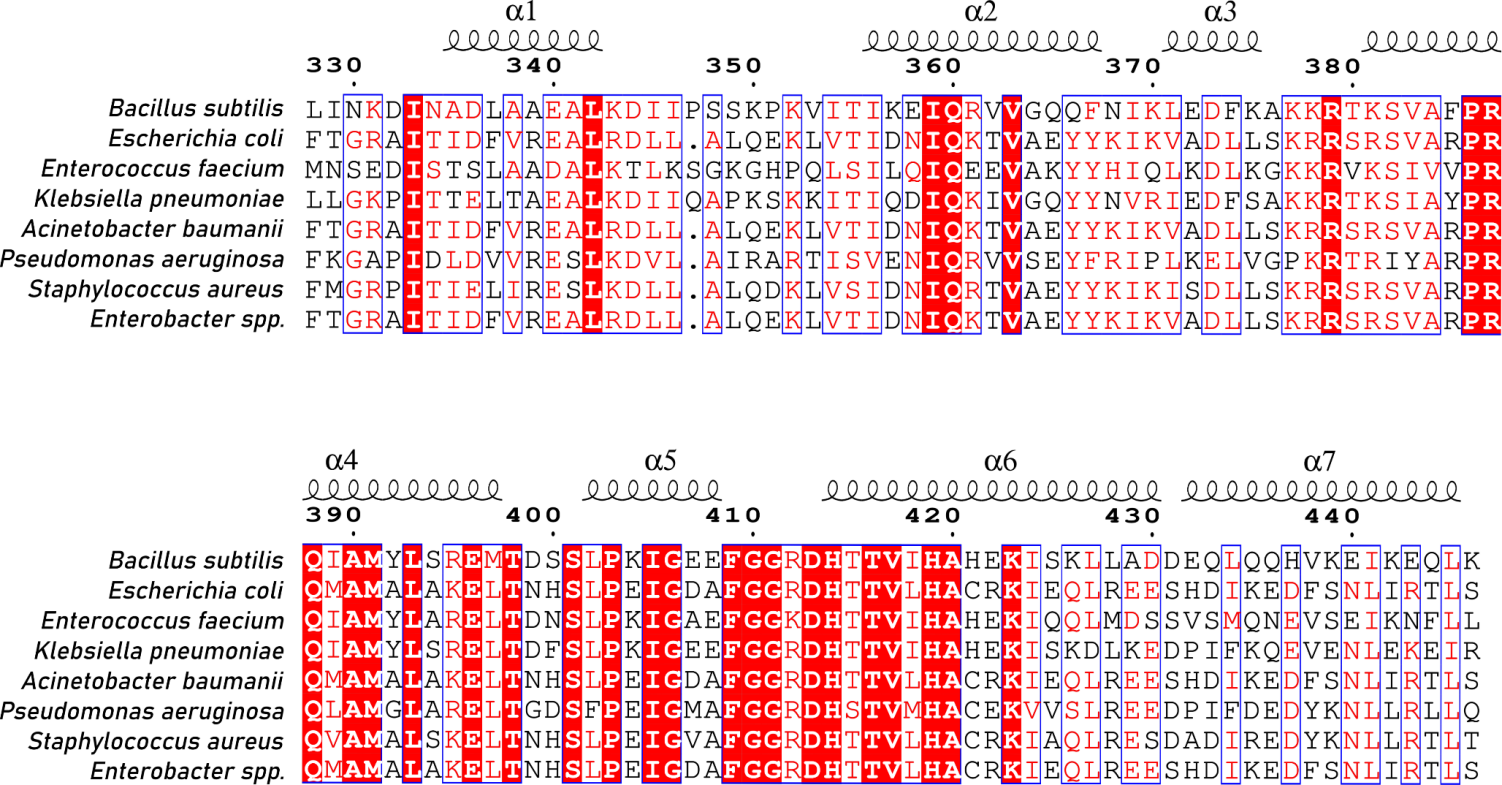
Sequence alignment of *Bacillus subtilis* DnaA^328–446^ to DnaA from high priority pathogens *Escherichia coli*, *Enterococcus faecium*, *Staphylococcus aureus*, *Klebsiella pneumoniae*, *Acinetobacter baumanii*, *Pseudomonas aeruginosa*, and *Enterobacter spp.* Residue numbers are consistent with *B. subtilis* DnaA. Secondary structural elements are indicated as reported in PDB: 8BTG (Pelliciari et al. 2023). This figure was generated by ESPript (Robert and Gouet 2014)

The functionality of DnaA domain IV in engaging DnaA-boxes in *oriC* is essential for DNA replication and therefore the cell cycle. Therefore, domain IV proves a potential target for the discovery of novel antibacterial therapeutics. Notably, domain IV has high sequence conservation – particularly in the basic loop and HTH motif regions that directly engage the DnaA-box (Roth and Messer 1995). Moreover, the DnaA-box consensus sequence is widely conserved across bacteria (Zyskind et al. 1983; Fuller et al. 1984). This conservation extends to the highest priority pathogens (Fig. 1) suggesting that an inhibitor of domain IV could have broad-spectrum therapeutic activity. In this study, we report the ^1^H, ^15^N, and ^13^C backbone and ^13^Cβ assignments of soluble DnaA^328–446^ from *B. subtilis* at 298 K in the presence of 20 mM sodium phosphate at pH 7.6 and 100 mM NaCl. This construct encompasses the N-terminal linker region and the entirety of domain IV.

## Methods and experiments

### Sequence alignment

The sequence of DnaA^328–446^ from *Bacillus subtilis* (UniProt: P05648) was aligned to a panel of high priority pathogens *Escherichia coli* (UniProt: P03004), *Enterococcus faecium* (UniProt: J6Z2M0), *Staphylococcus aureus* (UniProt: P68867), *Klebsiella pneumoniae* (UniProt: B5XT51), *Acinetobacter baumanii* (UniProt: A0A059ZTB3), *Pseudomonas aeruginosa* (Q02V80), and *Enterobacter spp.* (UniProt: A0A5Q2KA53) using MultAlin (Corpet 1988).

### Recombinant protein expression and purification

**Fig. 2:**
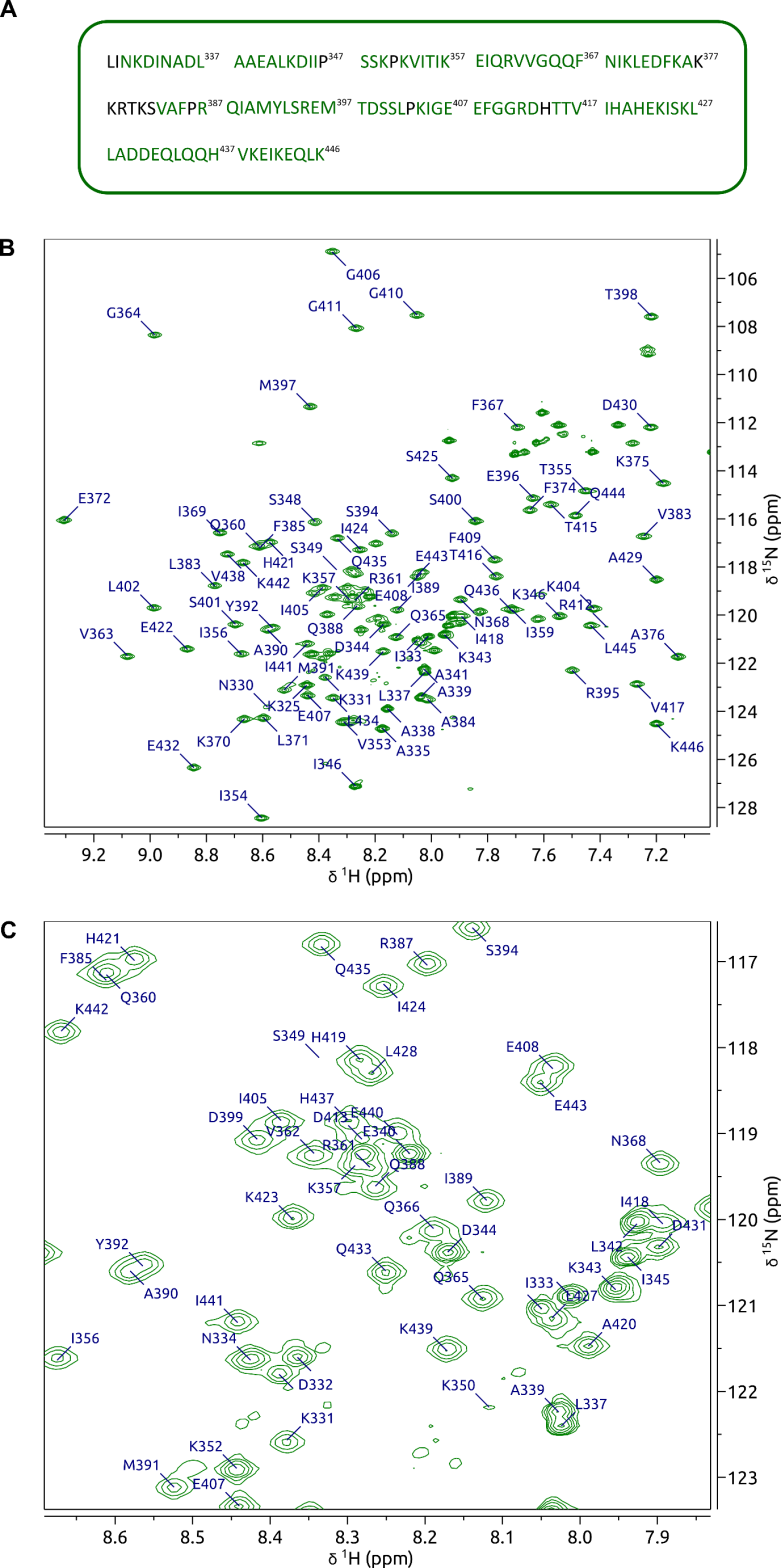
NMR assignment of DnaA^328–446^ from *B. subtilis* at 298 K in the presence of 20 mM sodium phosphate at pH 7.6 and 100 mM NaCl. (**A**) The amino acid sequence used in the assigned construct with assigned residues in green and unassigned residues in black. (**B**) Labelled 2D ^1^H,^15^N-HSQC of backbone DnaA^328–446^ signals at pH 7.6 in the presence of 20 mM sodium phosphate and 100 mM NaCl with (**C**) a zoomed view of the crowded region between 8.7 ppm and 7.8 ppm along the ^1^H-dimension

The construct of DnaA domain IV used in this study is based on the sequence from *Bacillus subtilis* (UniProt accession code P05648) encapsulating residues 328–446. The full construct amino acid sequence is displayed in Fig. 2A.

Double-labelled (^15^N, ^13^C) protein was expressed in M9 minimal media containing 4 g/L ^13^C-glucose and 0.5 g/L ^15^N-ammonium chloride. Plasmids encoding His_14_-SUMO-DnaA^328–446^ and chloramphenicol acetyltransferase were transformed into BL21(DE3)-pLysS. Cells were initially inoculated into 10 mL Luria Bertani (LB) medium supplemented with 100 µg/mL ampicillin and 30 µg/mL chloramphenicol and incubated overnight with shaking at 180 rpm and 37 °C. From the preculture, 100 mL LB medium supplemented with ampicillin and chloramphenicol was inoculated to OD_600_ ~ 0.05 and incubated with shaking at 180 rpm and 37 °C until OD_600_ ~ 1.3 was reached. The cells were harvested by centrifugation and washed once with M9 minimal medium to remove residual LB media. The cells were reharvested by centrifugation and resuspended into 1 L M9 minimal medium supplemented with ampicillin and chloramphenicol. The cells were incubated at 180 rpm and 37 °C until the OD_600_ reaches double the initial value. The temperature was then reduced to 30 °C before expression was induced through addition of isopropyl β-d-1-thiogalactopyranoside (IPTG) to 1mM. The cells were harvested 7 h post-induction by centrifugation and resuspended in 20 mL Buffer A (25 mM HEPES-KOH pH 7.6, 250 mM potassium glutamate, 30 mM imidazole) and flash frozen in liquid nitrogen for storage at − 80 °C until further use.

The cell pellet suspensions were thawed and supplemented with 1 EDTA-free protease inhibitor tablet (Roche – 1187358001), lysozyme (Merck – 62971) to 1 mg/mL, and DNase (Merck – 260913) to 50 µg/mL. Whilst placed on ice, the cells were disrupted by sonication (Sonics Vibra-cell, 25 min of 5 s ON, 20 s OFF cycles at 30% power). The lysate was clarified by centrifugation at 20,000 *g* for 30 min at 4 °C and passed through a 0.45 μm filter. The clarified lysate was applied to a pre-equilibrated 5mL HisTrap HP (Cytiva) column and washed with 100 mL Buffer A. The column was further washed with 100 mL of a high salt buffer (25 mM HEPES-KOH pH 7.6, 1 M potassium glutamate, 30 mM imidazole). Bound protein was eluted with Buffer B (25 mM HEPES-KOH pH 7.6, 250 mM potassium glutamate, 300 mM imidazole) over a 100 mL linear gradient (0-100%). Fractions containing tagged DnaA domain IV were pooled and concentrated to at least 15 mL (Amicon^®^ Ultra − 15). To remove the His_14_-SUMO tag, His_14_-TEV-SUMO protease (Frey and Görlich 2014) was added for digestion. 0.2 mL of a 6 mg/mL solution was added for each litre of expression media being purified. With the added protease, the sample was dialysed overnight against 500 mL Buffer A at 4 °C with agitation.

The following morning the sample was removed from the dialysis cassette and passed through a 0.2 μm filter. The sample was then re-applied to pre-equilibrated 5mL HisTrap HP column and the cleaved protein collected in the flowthrough. Fractions containing untagged DnaA domain IV were pooled and concentrated to 1 mL (Amicon^®^ Ultra − 4) at a final protein concentration of 700 µM. For NMR compatibility, the buffer was exchanged to 20 mM sodium phosphate pH 7.6, 100 mM NaCl through overnight dialysis at 4 °C with agitation. After buffer exchange, any precipitation was removed by centrifugation before flash freezing in liquid nitrogen.

### NMR experiments

Sequential backbone chemical shifts of *B. subtilis* DnaA^328–446^ were assigned based on numerous 2D and 3D NMR experiments. ^13^C^15^N-labelled protein was prepared in a 95% H_2_O/5% D_2_O solvent ratio to a final concentration of 370 µM in the presence of 20 mM sodium phosphate pH 7.6, 100 mM NaCl and contained in a shaped tube (Bruker: Z106898). ^1^H,^15^N-HSQC (Bodenhausen and Ruben 1980), HNCO, HN(CA)CO, HNCACB, and CBCA(CO)NH (Bax and Grzesiek 1993) experiments were collected at 298 K on Bruker Avance spectrometers operating at 800- or 950-MHz ^1^H frequency. Water-flipback was utilised in the^1^H,^15^N-HSQC. Non-uniform sampling (NUS) was employed for the 3D experiments with 25% sampling.

To characterise backbone protein dynamics, a {^1^H}-^15^N heteronuclear NOE (Zhu et al. 2000) experiment was collected at 298 K on a Bruker Avance spectrometer at 700-MHz ^1^H frequency. ^15^N-labelled protein was prepared in a 90% H_2_O/10% D_2_O solvent ratio to a final concentration of 250 µM in the presence of 20 mM sodium phosphate pH 7.6, 100 mM NaCl. Spectra with and without saturation of H_N_ resonances were collected as a pseudo-3D dataset. The proton saturation period was 4 s.

Reconstruction of the 3D HNCO, HN(CA)CO, HNCACB, and CBCA(CO)NH datasets were performed with SMILE (Ying et al. 2017). All spectra were processed using NMRPipe and NMRDraw (Delaglio et al. 1995), and analysed using CcpNmr AnalysisAssign (Skinner et al. 2016).

### Secondary structure prediction

The secondary structure elements were predicted using TALOS+ (Shen et al. 2009) by inputting ^1^H^N^, ^15^N^H^, ^13^Cα, and ^13^Cβ chemical shifts where assigned. TALOS + outputs the probabilities for each residue to be in an α-helix, *P(*α*)*; β-strand, *P(*β*)*; or loop regions, *P(L)*, all totalling to 1.0. These probabilities were collated into single a value as follows such that a values closer to 0 indicated a loop region, values closer to 1 indicated an α-helix, and values closer to − 1 a β-strand:

$$\:Structural\:Prediction\:=\:P\left(\alpha\:\right)-P\left(\beta\:\right)$$

### Extent of assignment and deposition

Overall, the backbone ^1^H^N^, ^15^N^H^, ^13^C’, ^13^Cα, and ^13^Cβ chemical shifts of 92% of non-proline residues were assigned (106 out of 115) (Fig. 2A). The 2D ^1^H,^15^N-HSQC spectrum of DnaA domain IV at pH 7.6 is typical of a folded protein demonstrated by the wide dispersion of signals along the amide proton dimension (Fig. 2B). D332 and I333 each have two corresponding cross peaks adjacent to one another indicating conformational exchange in the N-terminal region (Fig. 2B). There is one notable unassigned cross peak at 8.27 and 124.31 ppm in the ^1^H^N^ and ^15^N^H^ dimensions respectively. The ^13^Cα, and ^13^Cβ chemical shifts of this peak correspond closest to that of an aspartic acid or leucine residue. The spectra were searched extensively but connectivity to this cross peak could not be identified.

**Fig. 3:**
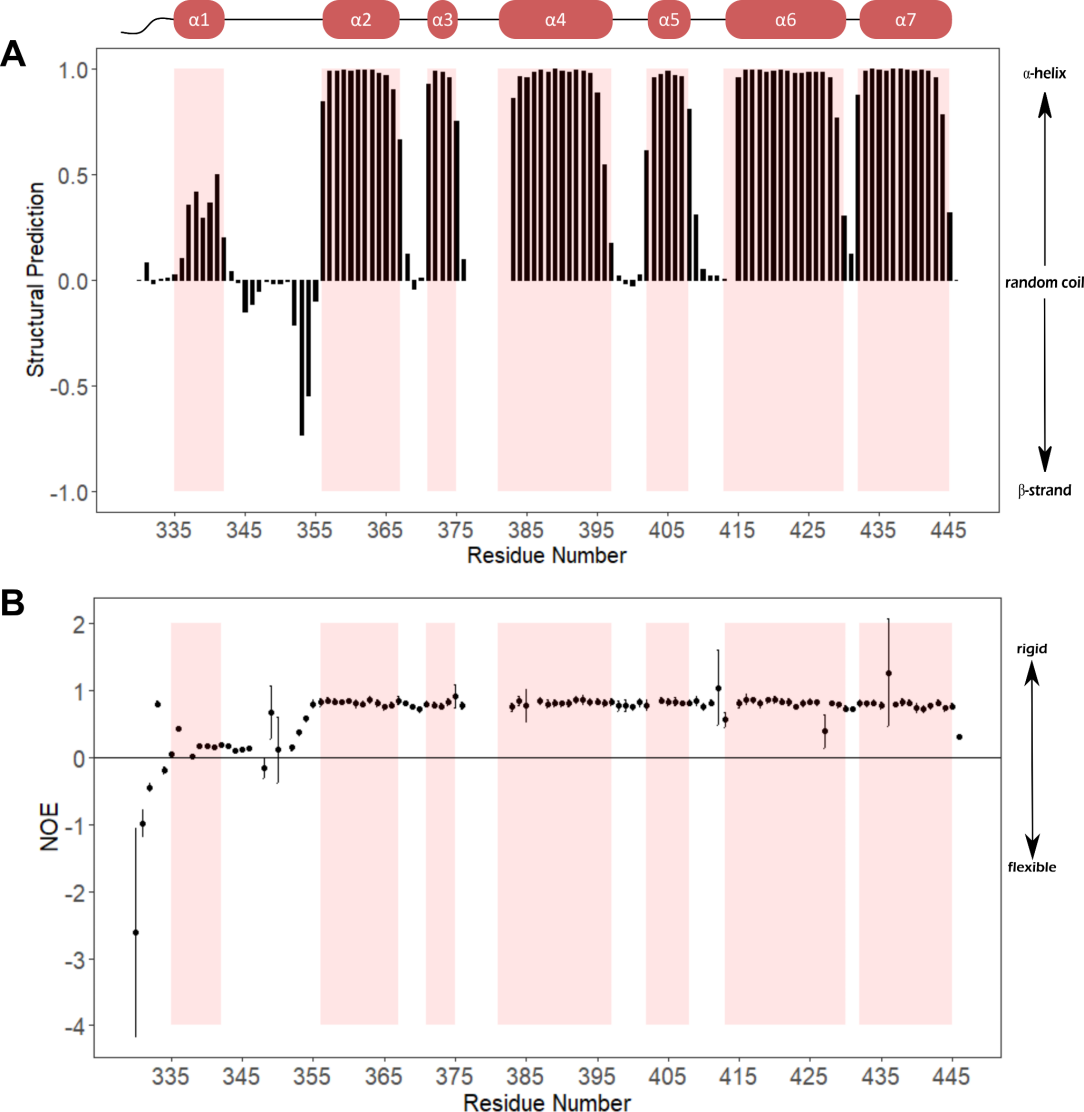
Secondary structure and dynamics of DnaA^328–446^ at pH 7.6 in the presence of 20 mM sodium phosphate and 100 mM NaCl. (**A**) The secondary structure predictions as derived by TALOS+ (Shen et al. 2009) and (**B**) {^1^H}-^15^N heteronuclear NOE measurements as a function of sequence number. High NOE (> 0.5) indicates a rigid structure whereas low NOE indicates flexibility. Secondary structural elements as reported in cryo-EM structure featuring domain IV PDB: 8BTG (Pelliciari et al. 2023) are displayed and shaded in red. Unassigned residues have no value

The chemical shift assignments were then translated into secondary structure predictions using TALOS+ (Shen et al. 2009) which predicts backbone torsion angles from NMR chemical shifts. In agreement with the deposited crystal and cryo-EM structures of DnaA domains III and IV (Fujikawa et al. 2003; Pelliciari et al. 2023), TALOS + predicts seven discrete helices in this construct. An overlay of the secondary structure indicated in the cryo-EM structure, PDB ID: 8BTG (Pelliciari et al. 2023), with the TALOS + structural predictions shows very close alignment from helix α2 to the C-terminal helix α7 (Fig. 3A).

Helix α1 lies in the linker region connecting domains III and IV and does not adopt a rigid fold in this construct (Fig. 3). As this construct only comprises the linker region and domain IV, this may be a consequence of removing this short helix from the context of domain III.

The secondary structural predictions, together with the {^1^H}-^15^N heteronuclear NOE data, demonstrate that the domain IV comprises a rigid fold of six α-helices (α2- α7) and four loops (Fig. 3). The secondary structure prediction does calculate a region of β-character in the N-terminal linker region between K352 and T355 just before α2. However, structural prediction in this region is of low confidence (Fig. S1) and the heteronuclear NOE data does not suggest these residues adopt a rigid fold.

Domain IV interacts with the minor groove of the DnaA-box through the basic loop N-terminal to α4 (Fujikawa et al. 2003). Interestingly, resonances for six consecutive residues in this region from K337 to S382 could not be assigned. Difficulties in extracting these amide proton and nitrogen resonances suggest the apo-state of this region is mobile and in intermediate-exchange. In addition, resonances could not be assigned for H414, a conserved N-terminal residue of α6 which inserts into the major-groove of the DnaA-box (Fujikawa et al. 2003; Pelliciari et al. 2023). Encouragingly, resonances for all remaining non-proline residues in the implicated region for DnaA-box binding are assigned. Last, the resonances for the two N-terminal amino-acids L328 and I329 could not be assigned, a possible result of rapid amide proton exchange with water. In total, the coverage of this assignment is sufficient to.

facilitate mechanistic studies regarding the linker between domains III and IV and the interaction between the domain IV and the DnaA-box with greater resolution possible for residues contacting the major-groove. This can be extended to facilitating the development of novel domain IV ligands and inhibitors.

## Electronic supplementary material

Below is the link to the electronic supplementary material.

Supplementary Material 1

## Data Availability

The reported NMR assignments of DnaA domain IV from Bacillus subtilis at pH 7.6 and 298 K are deposited in the Biological Magnetic Resonance Data Bank (BMRB) with the accession code 52533.
