# Supplementary material for: NMR assignment of the conserved bacterial DNA replication protein DnaA domain IV
Source: Biomol NMR Assign. 2024 Oct 4;18(2):315–21. doi: 10.1007/s12104-024-10206-1 (PMC11511705; doi:10.1007/s12104-024-10206-1)
Supplement: Supplementary file 1 — Supplementary Material 1 [file 12104_2024_10206_MOESM1_ESM.pdf]

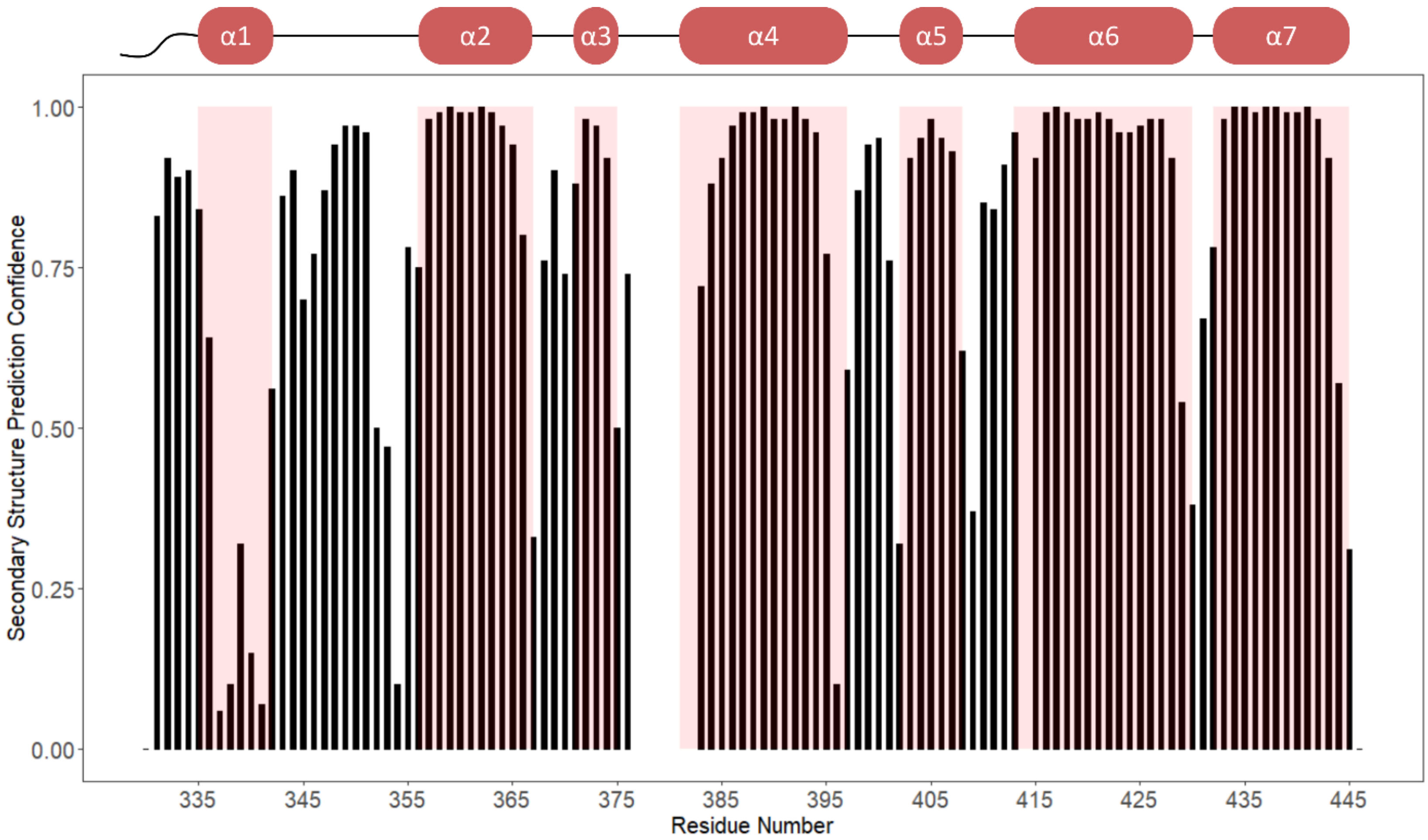

Figure S1. Confidence of secondary structure predictions of DnaA$^{328\text{-}446}$ at pH 7.6 in the presence of 20 mM sodium phosphate and 100 mM NaCl. The confidence of given predictions are defined as the difference between the probabilities of the two most favoured states (Shen et al. 2009). Unassigned residues have no value.
